# LGE of left atrial ablation lesions: effect of imaging time on lesion visualization

**DOI:** 10.1186/1532-429X-13-S1-P261

**Published:** 2011-02-02

**Authors:** Benjamin Knowles, Jeff Hsing, Warren J Manning, Dana C Peters

**Affiliations:** 1 Cardiovascular Division, Beth Israel Deconess Medical Center, Boston, MA, USA

## Introduction

Late gadolinium enhancement (LGE) of radiofrequency (RF) ablation lesions in the left atrium (LA) is an emerging tool in the assessment of pulmonary vein isolation (PVI) of atrial fibrillation patients. Ablation lesions, however, may exhibit different pharmacokinetics to that of a myocardial infarction. Furthermore, due to the thin LA myocardium, blood-lesion CNR is critical to visualization. Consequently, we sought to study the effect of imaging time on lesion visualization, in patients imaged 1 day post PVI with 3D LGE.

## Method

Nine patients were imaged 1 day post PVI on a 1.5T Philips Achieva system, with a 5-channel cardiac coil (Philips Healthcare, Best, The Netherlands). Patients underwent 2 or 3 3D LGE scans (mean = 2.4±0.7) at times ranging from 12-43 minutes post administration of 0.2mmol/kg of Gd-DTPA (Magnevist, Bayer Healthcare). Details on 3D LGE imaging sequence can be found in [1]. SNR and CNR (lesion-blood, and lesion-LV myocardium) measurements were made using ROIs in the anterior section of the left superior pulmonary vein (LSPV); the posterior wall and posterior section of the right superior pulmonary vein (RSPV). P-values were calculated from the correlation coefficients.

## Result and discussion

**Figure 1:**
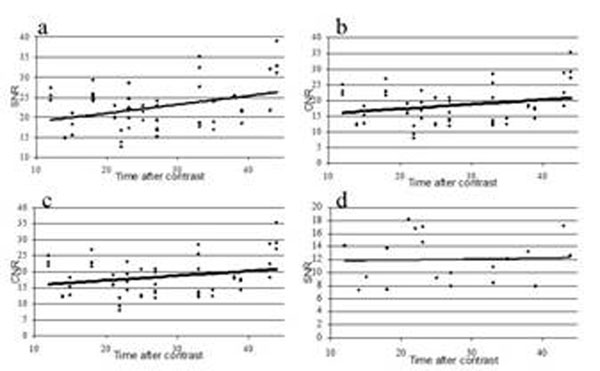
(a) Graph of CNR between LGE and the blood pool in the LA with increasing time (p=-.0004). (b) Graph of CNR between LGE and healthy myocardium in the LV with increasing time (p=0.06). (c) Graph showing the relationship of the SNR of LGE with increasing time (p=0.004). (d) Graph showing the relationship of the SNR of blood with increasing time after contrast administration.

**Figure 2:**
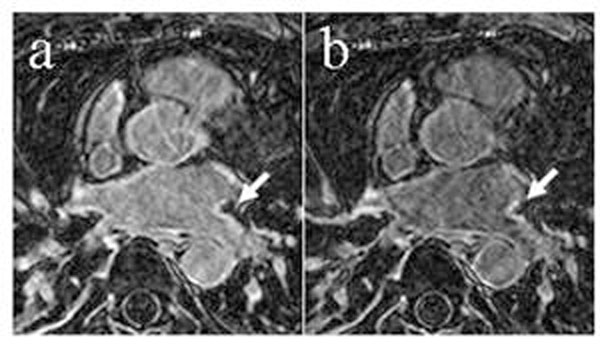
LGE images of the LA at 33 mins (a) and 43 mins (b) post contrast agent. The increase in contrast between the LGE and the blood is apparent (arrow)

SNR of lesions, and CNR of lesion-blood increased with increasing time after contrast administration (Figure 1a, c, both p<0.001), whereas the CNR of lesion-LV myocardium failed to provide significance for this study group (Figure 1b, p=0.06). The SNR of blood did not change significantly (p=0.85). Figure 2 shows LGE images at two times post injection. The findings suggest that contrast is retained in ablation lesions for a longer time than in the blood or healthy myocardium. Thus with increasing time, contrast between lesion and blood increases. The lack of increase in blood SNR and lesion-myocardium may be due to the inversion time, which optimally increases with time.

## Conclusion

In RF ablation lesion imaging using the LGE technique, it may be of benefit to image at later times in order to increase the CNR of the lesions with blood. The increase in CNR and SNR is likely to be due to the difference in the pharmacokinetics of the contrast agent in the lesion, in which uptake and wash-out are slower, compared to the pharmacokinetics inside the blood and healthy myocardium.
